# Autonomic Nervous System in the Control of Energy Balance and Body Weight: Personal Contributions

**DOI:** 10.1155/2013/639280

**Published:** 2013-04-11

**Authors:** G. Messina, V. De Luca, An. Viggiano, A. Ascione, T. Iannaccone, S. Chieffi, M. Monda

**Affiliations:** ^1^Department of Experimental Medicine, Section of Human Physiology and Clinical Dietetic Service, Second University of Naples, Via Costantinopoli 16, 80138 Naples, Italy; ^2^Faculty of Medicine, University of Salerno, Salerno, Italy; ^3^Faculty of Motor Sciences, University of Naples “Parthenope,” Naples, Italy

## Abstract

The prevalence of obesity is increasing in the industrialized world, so that the World Health Organization considers obesity as a “pandemia” in rich populations. The autonomic nervous system plays a crucial role in the control of energy balance and body weight. This review summarizes our own data and perspectives, emphasizing the influence exerted by autonomic nervous system on energy expenditure and food intake, which are able to determine the body weight. Activation of the sympathetic discharge causes an increase in energy expenditure and a decrease in food intake, while reduction of food intake and body weight loss determines a reduction of the sympathetic activity. On the other hand, pathophysiological mechanisms of the obesity involve alterations of the sympathetic nervous system in accordance with the “Mona Lisa Hypothesis,” an acronym for “most obesities known are low in sympathetic activity.” Furthermore, the parasympathetic influences on the energy expenditure are analyzed in this review, showing that an increase in parasympathetic activity can induce a paradoxical enhancement of energy consumption.

## 1. Introduction

Body weight stability and the associated regulatory processes depend upon nutrient intake, but are also influenced by compensatory genetic-dependent metabolic and neuroendocrine mechanisms [1–4].

The control of the maintenance of body composition has been the subject of a number of theories or pathways. Systems that control food intake and/or energy expenditure are able to influence body weight. Several substances are able to influence food intake. The “glucostatic hypothesis” emphasizes the role of blood glucose, considering that an increase in glucose blood level induces a reduction of food intake [5]. Leptin, a peptide secreted by white adipose tissue, acts on the hypothalamic areas inducing a reduction of food ingestion. This is in accord with the “lipostatic hypothesis” of food intake [6, 7]. Gastrointestinal hormones also lower food intake; this influence is known as the “hypothesis of gastrointestinal control of food intake” [8, 9]. The autonomic nervous system is involved in the control of eating behavior through influences exerted on the production and loss of heat [10, 11]. Thus, the control of body temperature is strictly associated with the control of body weight; this is in accord with the “thermoregulatory hypothesis” of food intake [12].

On the other hand, the metabolic balance is controlled by the autonomic nervous system, so that the vegetative influences affect the storage and the consumption of energy.

Adipose tissue acts as an endocrine organ by producing various signalling cytokines called adipokines (including leptin, free fatty acids, tumour necrosis factor-*α*, interleukin-6, C-reactive protein, angiotensinogen, and adiponectin). A chronic dysregulation of certain adipokines can have deleterious effects on insulin signalling. Chronic sympathetic overactivity is also known to be present in central obesity, and recent findings demonstrate the consequence of an elevated sympathetic outflow to organs such as the heart, kidneys, and blood vessels. Chronic sympathetic nervous system overactivity can also contribute to a further decline of insulin sensitivity, creating a vicious cycle that may contribute to the development of the metabolic syndrome and hypertension. The cause of this overactivity is not clear, but may be driven by certain adipokines [13]. Furthermore, the postprandial activation of the peripheral sympathetic nervous system is crucial to maintain energy balance. A contribution of postprandial sympathetic activation to the thermic effect of food is not always evident and depends on the size and composition of the meal, with carbohydrates having the clearest effect. Signals related to food intake from various origins (e.g., gut, hepatoportal area, baroreceptors) are integrated in the brain and result in increased peripheral sympathetic outflow. It is of interest to emphasize the role of diet composition (according to the life style of subjects) in the level of sympathetic activation during the day in view of the potential role of adrenergic overactivity in the pathogenesis of obesity and its metabolic syndrome [14].

Although it is reported that low-frequency band (LF-HRV) represents a noninvasive marker of sympathetic activity, there are recent studies which report that this assumption is controversial. LF power may correlate more with baroreflex function and/or stress that with the cardiac sympathetic innervations [15, 16]. This vision should modify the interpretations about the sympathetic function in the pathophysiology of the obesity.

Since the incidence of body weight superior to normal values is increasing in the industrialized world, the World Health Organization considers obesity as a “pandemia” in rich populations. Investigation into the mechanisms that control body weight give growing relevance to the possibilities of new strategies to reduce the incidences of overweight and obesity, which are frequently associated with metabolic and cardiovascular diseases.

This review reports our evidences showing that the autonomic nervous system controls body weight by influencing food intake and energy consumption. The general research project was to test influence of the autonomic nervous system on energy balance under various conditions, which change the sympathetic and/or parasympathetic activities.

## 2. Experimental Evidences

### 2.1. Animal Studies

**Scheme 1:**
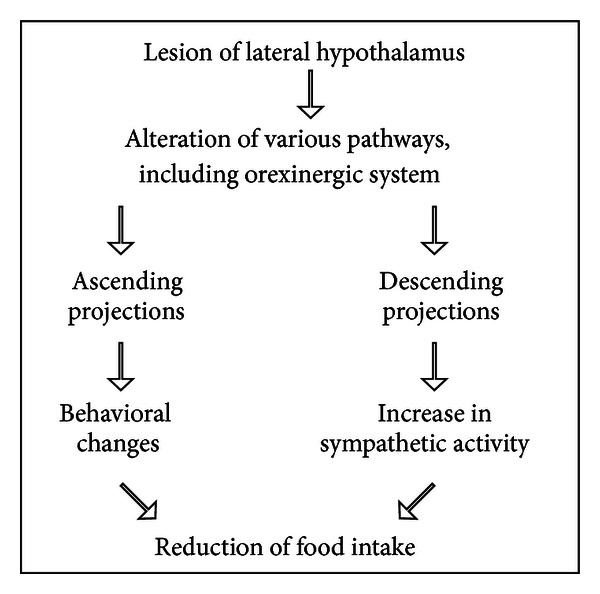
Changes in eating behavior induced by lesion of the lateral hypothalamus.

The effect of intraperitoneal injection of lysine acetylsalicylate was tested on (1) food intake and (2) the sympathetic enhancement induced by lesion of the lateral hypothalamus. Lysine acetylsalicylate modifies the aphagia by increasing food intake, and it reduces the enhancements of the sympathetic discharge induced by the lateral hypothalamic lesion [17]. The electrolytic lesion in the lateral hypothalamus regulates body weight at a lower level. The lesioned rats lose body weight at a faster rate than sham-lesioned controls subjected to the same degree of food deprivation [18]. This experiment confirms that an increase in the sympathetic activity reduces food intake (see Scheme 1), in accord with the findings of Bray [19]. On the other hand, these data show that an inhibitor of prostaglandin synthesis can modify the aphagia induced by the lesion of the lateral hypothalamus, throughout a reduction of the sympathetic discharge.

**Scheme 2:**
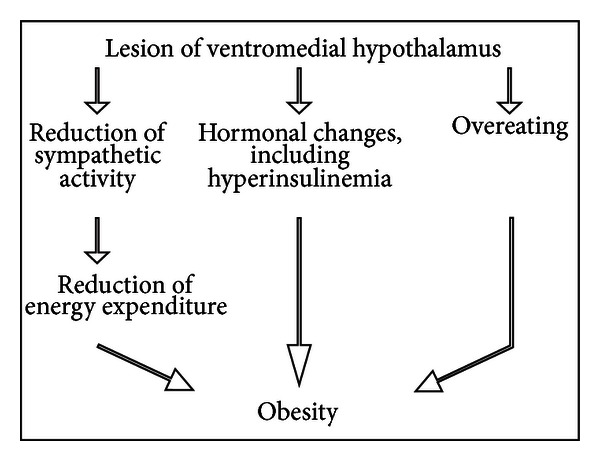
Changes in energy expenditure induced by lesion of the ventromedial hypothalamus.

The firing rate of sympathetic nerves to interscapular brown adipose tissue (IBAT) was monitored both before and after 5 g of food intake in 24 h fasted rats with a lesion of the ventromedial hypothalamus and in 24 h fasted rats with sham-lesion. The firing rate of nerves to IBAT increased after food intake in sham-lesioned rats. This increase was significantly reduced in the lesioned rats. These findings indicate that the sympathetic nervous system is involved in the postingestional activation of the sympathetic discharge, and reduction of this activation occurs when the ventromedial hypothalamus is lesioned [20]. A long-term reduction of postingestional thermogenesis may contribute to the obesity induced by this hypothalamic lesion through a decrease in energy expenditure induced by reduced sympathetic activity (see Scheme 2). Since there is a close relationship between the sympathetic activity and food intake [19], a reduction in the sympathetic response after food intake could induce an increase of total amount of ingested food. This reduction may be another factor in the induction of obesity due to ventromedial hypothalamic lesions. In other words, the increased body weight may be caused by a reduction of satiety signals and a decrease in postingestional energy expenditure.

**Scheme 3:**
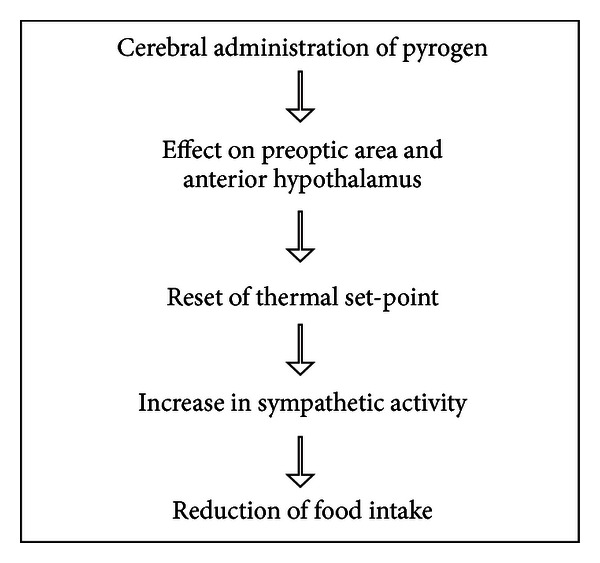
Changes in eating behavior induced by pyrogen injection.

The firing rate of the sympathetic nerves to interscapular brown adipose tissue and food intake were monitored in 24 h fasting male Sprague-Dawley rats before and after food presentation. Pyrogen (500 ng of prostaglandin E_1_) or saline was injected into the lateral cerebral ventricle immediately before food presentation. The increase in the sympathetic discharge due to prostaglandin E_1_ is associated with a decrease in food intake [21]. The simultaneous measurement of the sympathetic firing rate and food intake is the nicest demonstration of the feedback between the sympathetic nervous system and food intake. The sympathetic activity rises before food intake terminates. This implies that the rise in sympathetic discharge serves as an endogenous satiety signal (see Scheme 3).

### 2.2. Human Studies

**Scheme 4:**
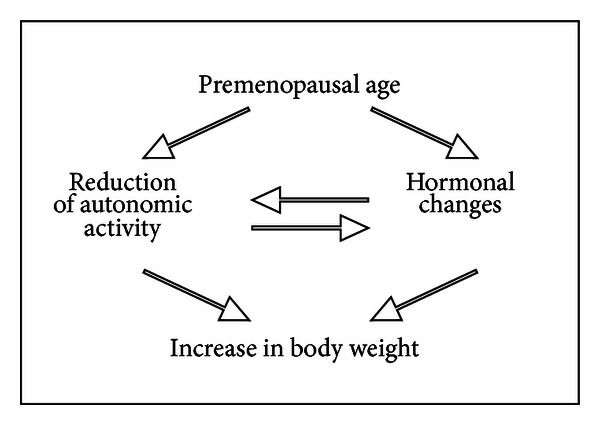
Body weight gain during premenopausal age.

Vegetative modulation, expressed as heart rate variability (HRV) power spectral analysis, was analyzed in lean and obese women at premenopausal or postmenopausal age. The HRV-power spectrum was evaluated on a 5-min long ECG recording. The absolute values of the spectrum were summed in the following frequencies: a low frequency (0.04–0.15 Hz; LF) and high frequency (0.15–0.40; HF) range. LF and HF were values used to estimate the sympathetic and parasympathetic activity. LF and HF values of premenopausal obese women were lower than values of lean women. In postmenopause, LF and HF have a similar decrease in lean and obese women, as a consequence no difference can be found. These findings indicate a reduction of the vegetative modulation in obese young women and the reduction of the autonomic control regards both the sympathetic and parasympathetic components [22]. The reduction of the sympathetic branch could be an important factor in the maintenance of obesity in premenopausal age (see Scheme 4). Indeed, a reduction in the sympathetic activity could be related to a low energy expenditure, so that a reduced energetic cost could explain the higher body weight in premenopausal women. This vision is in accordance with the “Mona Lisa Hypothesis,” an acronym for “most obesities known are low in sympathetic activity” [23]. In this experiment, the autonomic activity of postmenopausal women is lower than that of premenopausal subjects. This indicates that the modifications of the autonomic modulation cannot be included among factors related to obesity in postmenopausal subjects. Many experimental evidence have demonstrated that an increase in sympathetic and thermogenic activity reduces food intake. Therefore, the obesity can be due to an increase in food intake associated to a reduced activity of the sympathetic nervous system. On the other hand, some study revealed lower respiratory sinus arrhythmia, as evaluated by the HF-HRV spectral analysis combined with deep breathing tests, which points to the presence of cardiac vagal dysfunction in obese adolescents [24]. Importantly, autonomic imbalance with decreased parasympathetic activity maybe the final common pathway in numerous conditions associated with increased morbidity and mortality [25]. The evaluation of cardiorespiratory interactions, in particular the heart rate variability, can provide diagnostic information about early subclinical autonomic dysfunction in obesity.

**Scheme 5:**
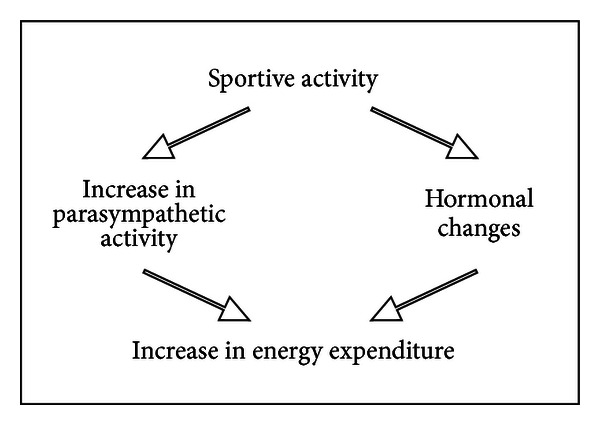
Changes in energy expenditure induced by sport.

Sports are known to induce several adaptive modifications, including changes in the activity of the autonomic nervous system and in resting energy expenditure (REE) [26, 27]. The parasympathetic tone is enhanced by physical training, so that a reduction in the heart rate (induced by vagal influence) is considered as an index of training status in athletes [28]. Since there are few studies concerning the comparison between vegetative and energetic changes of sedentary individuals and those of sportive subjects, the influence exerted by sedentary and basketball exercise training on the relationship between the activity of the autonomic nervous system and REE was evaluated. REE, body composition, and the level of activity of the autonomic nervous system were measured before and after a period of six months. The physical activity induced an increase in REE and free fat mass without variations in body weight. Basketball players showed a significant increase in the parasympathetic activity, measured by the power spectral analysis (PSA) of the heart rate variability (HRV). These findings (see Scheme 5) demonstrate that REE is higher in the athletes than in sedentary women, despite the augmented parasympathetic activity that is usually related to lower energy expenditure [29]. This is the first study to examine the effect of long-term training on relationship among cardiac HRV, REE, and body composition. In this study, an increase of the HF of the HRV-PSA has been noted in sportive women, confirming that exercise induces an increase of the parasympathetic activity at resting. On the contrary, the LF of the PSA of HRV has not been modified by sport activity, indicating that the basketball does not modify the sympathetic discharge. The increase in the parasympathetic activity is associated with an increase in REE. This association is an important result considering that the parasympathetic activity has generally been demonstrated to have an inverse relation to REE [30, 31].

## 3. Discussion and Conclusion

The above-reported evidences indicate that the autonomic nervous system can be considered a fundamental factor in the regulation of food intake and body weight. The sympathetic influence on this regulation is exerted by an influence on body temperature; this is in accord with the “thermoregulatory hypothesis” of food intake. The consequences of this hypothesis are that subjects with a high set-point of body temperature and/or low sympathetic activity are induced to eat a high quantity of food to elevate the sympathetic discharge and body temperature. Conversely, subjects with a low thermal set-point and/or a high sympathetic tone need to introduce a lower quantity of food to reach a prefixed thermal set-point. On the other hand, alterations of postprandial thermogenesis due to a reduced response of sympathetic activation can play an important role in inducing obesity. In other words, subjects with a low postprandial sympathetic activation need to introduce a higher quantity of foot to reach a prefixed body temperature. Our findings support the “Mona Lisa hypothesis” [23], which suggests that most types of obesity are due to an alteration of the sympathetic activity. On the other hand, being overweight increases the sympathetic discharge that contributes to induce pathologies related to abnormal body weight [32–34]. Furthermore, an increase in parasympathetic activity is able to enhance energy expenditure in sportive subjects. This indicates that not only the sympathetic activation but also the parasympathetic activation is able to increase energy expenditure, that in turn influences body weight. Further studies should be addressed to reveal new aspects of the control exerted by the autonomic nervous system on body weight, so that innovative strategies could be used for the prevention and therapy of obesity.

On the other hand, other factors other than the “Mona Lisa hypothesis” must be considered in the genesis of obesity. The “lipostatic hypothesis” indicates that adipokines such as leptin could play a crucial role in the gain of body weight. A reduction in leptin level signals to the brain to increase feeding and decrease energy expenditure. Leptin is an important factor linking energy stores to eating behavior [35]. The “hypothesis of gastrointestinal hormones” suggests that gut hormones are implicated in the control of body weight. These hormones regulate appetite, energy expenditure, and glucose homeostasis. They can act either via the circulation at target peripheral tissues, by activation of the vagus nerve, or by acting on key brain regions implicated in energy homeostasis such as the hypothalamus and brainstem [36, 37]. In conclusion, all factors of reported hypotheses can cooperate synergically to induce body weight alteration [38].
